# Supplementary material for: Regional Cerebral Oxygen Saturation and Risk of Delirium: A Systematic Review and Meta-Analysis
Source: Diseases. 2025 Nov 25;13(12):383. doi: 10.3390/diseases13120383 (PMC12731443; doi:10.3390/diseases13120383)
Supplement: Supplementary file 1 [file diseases-13-00383-s001.zip › diseases-3950206-supplementary.pdf]

a

Pooled mean baseline in Delirium & Monitor

Study or Subgroup	Baseline Pooled Delirium	SE	Delirium Total	Weight	Baseline Pooled Delirium IV, Random, 95% CI
**1.8.1 INVOS**					
Ahn 2021	55.3	0.75	230	7.4%	55.30 [53.83, 56.77]
Bennet 2021	54	2.85	14	6.5%	54.00 [48.41, 59.59]
Chen 2024	58.2	1.01	47	7.3%	58.20 [56.22, 60.18]
Fischer-Kumbruch 2022	64.8	2.31	13	6.8%	64.80 [60.27, 69.33]
Lim 2020	54.8	0.75	105	7.4%	54.80 [53.33, 56.27]
Schoen 2011	58.1	0.98	62	7.3%	58.10 [56.18, 60.02]
Soh 2016	67	9.1	9	3.1%	67.00 [49.16, 84.84]
Soh 2020	56	6	16	4.6%	56.00 [44.24, 67.76]
Susano 2021	61	1.06	53	7.3%	61.00 [58.92, 63.08]
Tobar 2018	72	0.39	26	7.4%	72.00 [71.24, 72.76]
**Subtotal (95% CI)**			**575**	**65.2%**	**59.91 [53.96, 65.87]**
Heterogeneity: Tau² = 82.99; Chi² = 795.48, df = 9 (P < 0.00001); I² = 99%					
Test for overall effect: Z = 19.73 (P < 0.00001)					
**1.8.2 FORESIGHT**					
Chan 2019	67.4	2.47	24	6.7%	67.40 [62.56, 72.24]
Cui 2021	69.3	0.93	35	7.3%	69.30 [67.48, 71.12]
Wood 2017	63.5	3.44	19	6.2%	63.50 [56.76, 70.24]
**Subtotal (95% CI)**			**78**	**20.3%**	**68.05 [65.30, 70.79]**
Heterogeneity: Tau² = 2.28; Chi² = 2.98, df = 2 (P = 0.23); I² = 33%					
Test for overall effect: Z = 48.51 (P < 0.00001)					
**1.8.3 Other**					
Mailhot 2019	66	0.64	173	7.4%	66.00 [64.75, 67.25]
Song 2022	69.9	1.83	16	7.1%	69.90 [66.31, 73.49]
**Subtotal (95% CI)**			**189**	**14.5%**	**67.57 [63.82, 71.32]**
Heterogeneity: Tau² = 5.73; Chi² = 4.05, df = 1 (P = 0.04); I² = 75%					
Test for overall effect: Z = 35.32 (P < 0.00001)					
**Total (95% CI)**			**842**	**100.0%**	**62.47 [58.40, 66.55]**
Heterogeneity: Tau² = 57.98; Chi² = 832.82, df = 14 (P < 0.00001); I² = 98%					
Test for overall effect: Z = 30.04 (P < 0.00001)					
Test for subgroup differences: Chi² = 6.08, df = 2 (P = 0.05), I² = 67.1%					

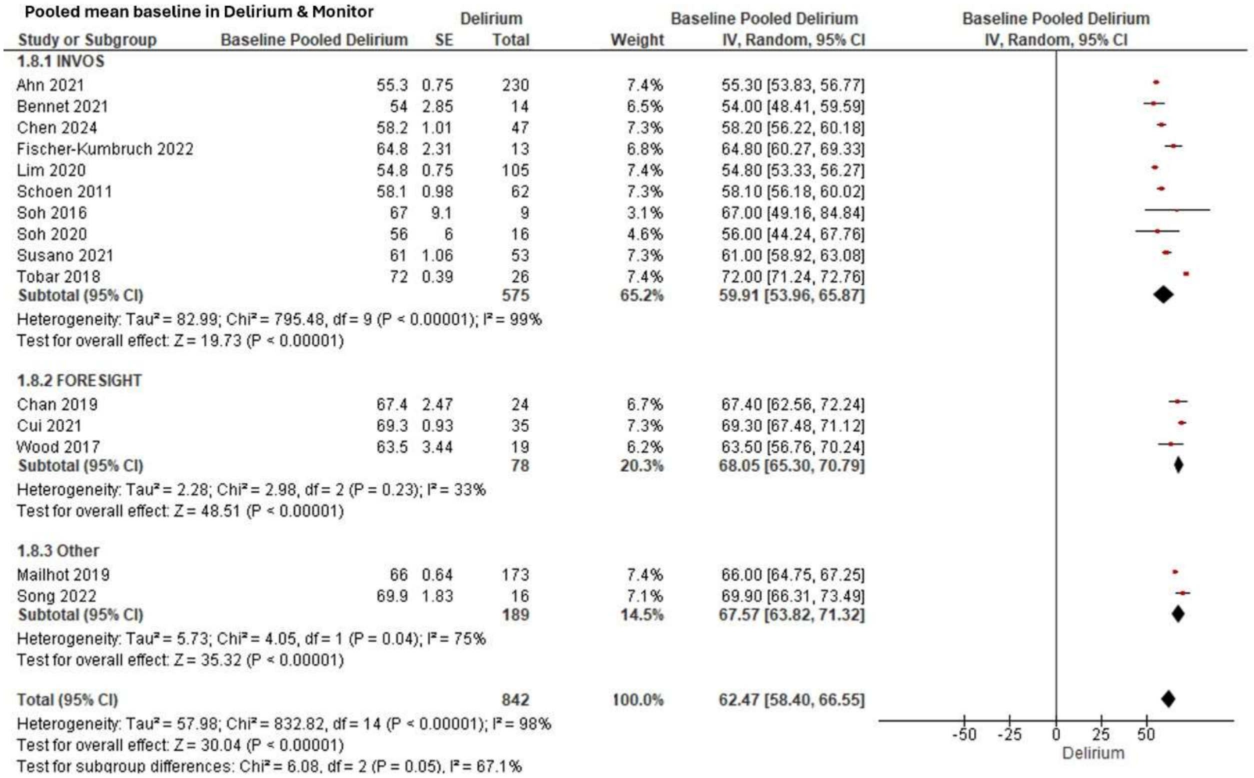

b

Pooled mean baseline in No Delirium & Monitor

Study or Subgroup	Baseline Pooled No Delirium	SE	No Delirium Total	Weight	Baseline Pooled No Delirium IV, Random, 95% CI
**1.11.1 INVOS**					
Ahn 2021	61.9	0.48	460	7.2%	61.90 [60.96, 62.84]
Bennet 2021	56.5	0.87	152	7.1%	56.50 [54.79, 58.21]
Chen 2024	60.5	0.84	85	7.1%	60.50 [58.85, 62.15]
Fischer-Kumbruch 2022	69.9	0.93	67	7.1%	69.90 [68.08, 71.72]
Lim 2020	55.5	0.41	710	7.2%	55.50 [54.70, 56.30]
Schoen 2011	63.1	0.55	169	7.2%	63.10 [62.02, 64.18]
Soh 2016	63	2.74	100	5.8%	63.00 [57.63, 68.37]
Soh 2020	62	0.71	97	7.1%	62.00 [60.61, 63.39]
Susano 2021	65	0.57	185	7.2%	65.00 [63.88, 66.12]
Tobar 2018	62	7.07	2	2.7%	62.00 [48.14, 75.86]
**Subtotal (95% CI)**			**2027**	**65.7%**	**61.89 [59.02, 64.76]**
Heterogeneity: Tau² = 18.87; Chi² = 367.93, df = 9 (P < 0.00001); I² = 98%					
Test for overall effect: Z = 42.24 (P < 0.00001)					
**1.11.2 FORESIGHT**					
Chan 2019	65.8	1.32	84	6.9%	65.80 [63.21, 68.39]
Cui 2021	70.4	0.47	140	7.2%	70.40 [69.48, 71.32]
Wood 2017	69	2.53	79	6.0%	69.00 [64.04, 73.96]
**Subtotal (95% CI)**			**303**	**20.0%**	**68.48 [65.07, 71.88]**
Heterogeneity: Tau² = 6.89; Chi² = 10.90, df = 2 (P = 0.004); I² = 82%					
Test for overall effect: Z = 39.44 (P < 0.00001)					
**1.11.3 Other**					
Mailhot 2019	69	0.64	173	7.2%	69.00 [67.75, 70.25]
Song 2022	69.3	0.74	85	7.1%	69.30 [67.85, 70.75]
**Subtotal (95% CI)**			**258**	**14.3%**	**69.13 [68.18, 70.08]**
Heterogeneity: Tau² = 0.00; Chi² = 0.09, df = 1 (P = 0.76); I² = 0%					
Test for overall effect: Z = 142.81 (P < 0.00001)					
**Total (95% CI)**			**2588**	**100.0%**	**64.24 [61.33, 67.15]**
Heterogeneity: Tau² = 30.42; Chi² = 886.98, df = 14 (P < 0.00001); I² = 98%					
Test for overall effect: Z = 43.25 (P < 0.00001)					
Test for subgroup differences: Chi² = 22.01, df = 2 (P < 0.0001), I² = 90.9%					

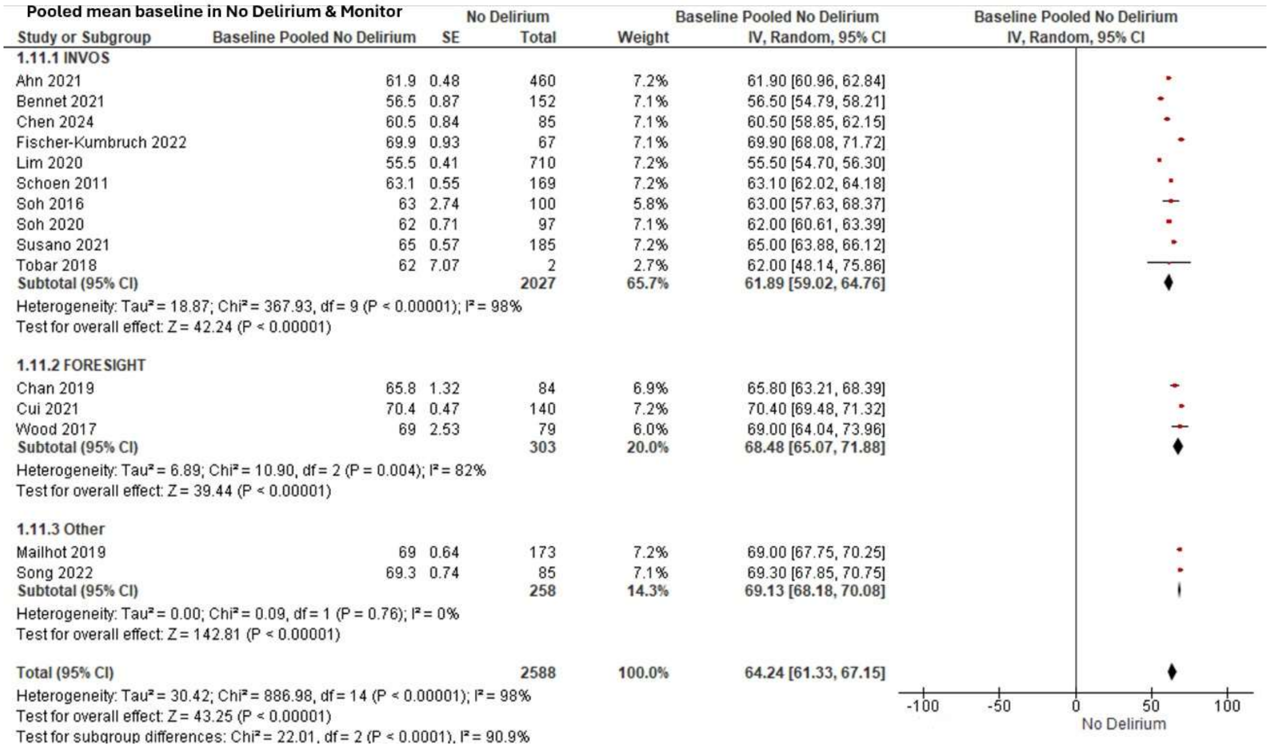

Figure S1. Mean baseline cerebral oxygen saturation values for (**a**) delirium and (**b**) non-delirium according to type of monitor

a

Pooled mean baseline in Delirium & Surgery

Study or Subgroup	Baseline Pooled Delirium	SE	Delirium Total	Weight	Baseline Pooled Delirium IV, Random, 95% CI
**2.2.1 Cardiac**					
Ahn 2021	55.3	0.75	230	7.4%	55.30 [53.83, 56.77]
Bennet 2021	54	2.85	14	6.5%	54.00 [48.41, 59.59]
Chan 2019	67.4	2.47	24	6.7%	67.40 [62.56, 72.24]
Chen 2024	58.2	1.01	47	7.3%	58.20 [56.22, 60.18]
Lim 2020	54.8	0.75	105	7.4%	54.80 [53.33, 56.27]
Mailhot 2019	66	0.64	173	7.4%	66.00 [64.75, 67.25]
Schoen 2011	58.1	0.98	62	7.3%	58.10 [56.18, 60.02]
Soh 2020	56	6	16	4.6%	56.00 [44.24, 67.76]
**Subtotal (95% CI)**			**671**	**54.7%**	**58.89 [54.95, 62.82]**
Heterogeneity: Tau² = 27.87; Chi² = 191.21, df = 7 (P < 0.00001); I² = 96%					
Test for overall effect: Z = 29.32 (P < 0.00001)					
**2.2.2 Abdominal**					
Fischer-Kumbruch 2022	64.8	2.31	13	6.8%	64.80 [60.27, 69.33]
Song 2022	69.9	1.83	16	7.1%	69.90 [66.31, 73.49]
Tobar 2018	72	0.39	26	7.4%	72.00 [71.24, 72.76]
**Subtotal (95% CI)**			**55**	**21.3%**	**69.41 [65.53, 73.28]**
Heterogeneity: Tau² = 9.21; Chi² = 10.48, df = 2 (P = 0.005); I² = 81%					
Test for overall effect: Z = 35.07 (P < 0.00001)					
**2.2.3 Other**					
Cui 2021	69.3	0.93	35	7.3%	69.30 [67.48, 71.12]
Soh 2016	67	9.1	9	3.1%	67.00 [49.16, 84.84]
Susano 2021	61	1.06	53	7.3%	61.00 [58.92, 63.08]
Wood 2017	63.5	3.44	19	6.2%	63.50 [56.76, 70.24]
**Subtotal (95% CI)**			**116**	**23.9%**	**64.92 [58.82, 71.02]**
Heterogeneity: Tau² = 27.98; Chi² = 35.06, df = 3 (P < 0.00001); I² = 91%					
Test for overall effect: Z = 20.85 (P < 0.00001)					
**Total (95% CI)**			**842**	**100.0%**	**62.47 [58.40, 66.55]**
Heterogeneity: Tau² = 57.98; Chi² = 832.82, df = 14 (P < 0.00001); I² = 98%					
Test for overall effect: Z = 30.04 (P < 0.00001)					
Test for subgroup differences: Chi² = 13.95, df = 2 (P = 0.0009), I² = 85.7%					

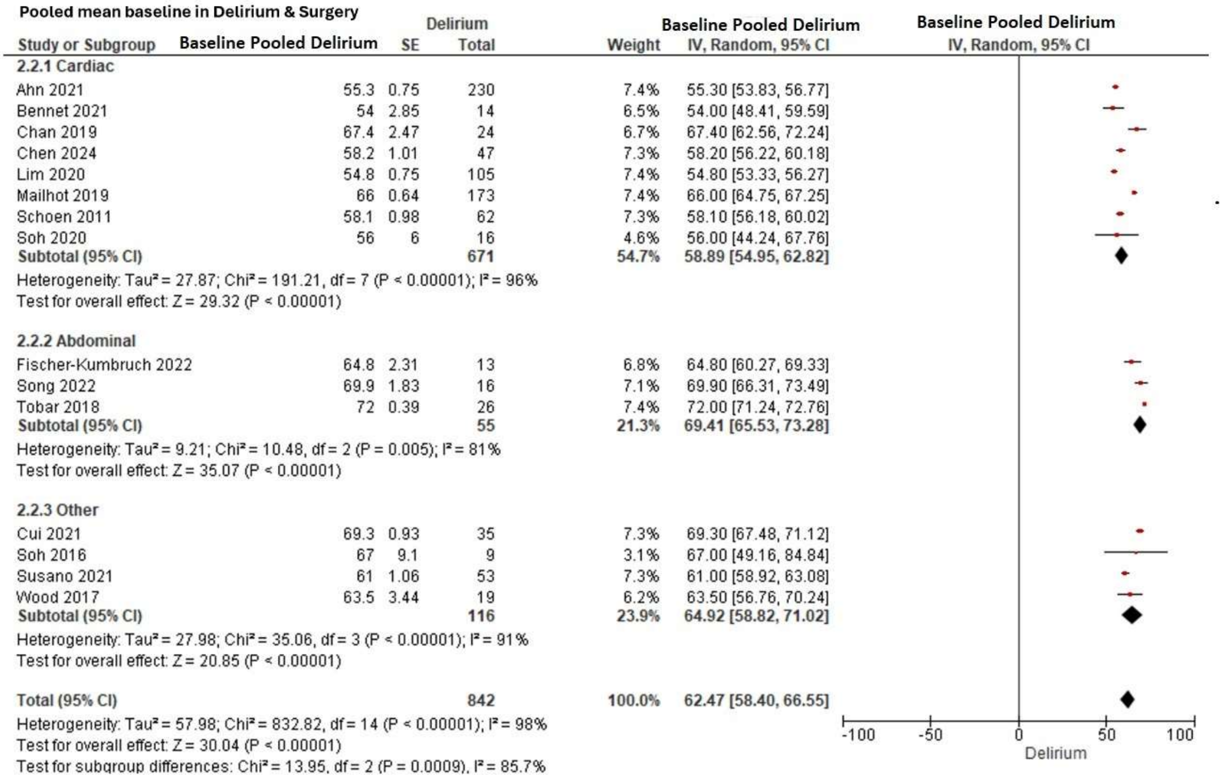

b

Pooled mean baseline in No Delirium & Surgery

Study or Subgroup	Baseline Pooled No Delirium	SE	No Delirium Total	Weight	Baseline Pooled No Delirium IV, Random, 95% CI
**2.3.1 Cardiac**					
Ahn 2021	61.9	0.48	460	7.2%	61.90 [60.96, 62.84]
Bennet 2021	56.5	0.87	152	7.1%	56.50 [54.79, 58.21]
Chan 2019	65.8	1.32	84	6.9%	65.80 [63.21, 68.39]
Chen 2024	60.5	0.84	85	7.1%	60.50 [58.85, 62.15]
Lim 2020	55.5	0.41	710	7.2%	55.50 [54.70, 56.30]
Mailhot 2019	69	0.64	173	7.2%	69.00 [67.75, 70.25]
Schoen 2011	63.1	0.55	169	7.2%	63.10 [62.02, 64.18]
Soh 2020	62	0.71	97	7.1%	62.00 [60.61, 63.39]
**Subtotal (95% CI)**			**1930**	**56.9%**	**61.76 [58.47, 65.06]**
Heterogeneity: Tau² = 22.06; Chi² = 395.76, df = 7 (P < 0.00001); I² = 98					
Test for overall effect: Z = 36.70 (P < 0.00001)					
**2.3.2 Abdominal**					
Fischer-Kumbruch 2022	69.9	0.93	67	7.1%	69.90 [68.08, 71.72]
Song 2022	69.3	0.74	85	7.1%	69.30 [67.85, 70.75]
Tobar 2018	62	7.07	2	2.7%	62.00 [48.14, 75.86]
**Subtotal (95% CI)**			**154**	**16.9%**	**69.48 [68.35, 70.61]**
Heterogeneity: Tau² = 0.00; Chi² = 1.38, df = 2 (P = 0.50); I² = 0%					
Test for overall effect: Z = 120.39 (P < 0.00001)					
**2.3.3 Other**					
Cui 2021	70.4	0.47	140	7.2%	70.40 [69.48, 71.32]
Soh 2016	63	2.74	100	5.8%	63.00 [57.63, 68.37]
Susano 2021	65	0.57	185	7.2%	65.00 [63.88, 66.12]
Wood 2017	69	2.53	79	6.0%	69.00 [64.04, 73.96]
**Subtotal (95% CI)**			**504**	**26.2%**	**67.06 [63.12, 71.00]**
Heterogeneity: Tau² = 13.22; Chi² = 57.10, df = 3 (P < 0.00001); I² = 95%					
Test for overall effect: Z = 33.36 (P < 0.00001)					
**Total (95% CI)**			**2588**	**100.0%**	**64.24 [61.33, 67.15]**
Heterogeneity: Tau² = 30.42; Chi² = 886.98, df = 14 (P < 0.00001); I² = 98%					
Test for overall effect: Z = 43.25 (P < 0.00001)					
Test for subgroup differences: Chi² = 19.43, df = 2 (P < 0.0001), I² = 89.7%					

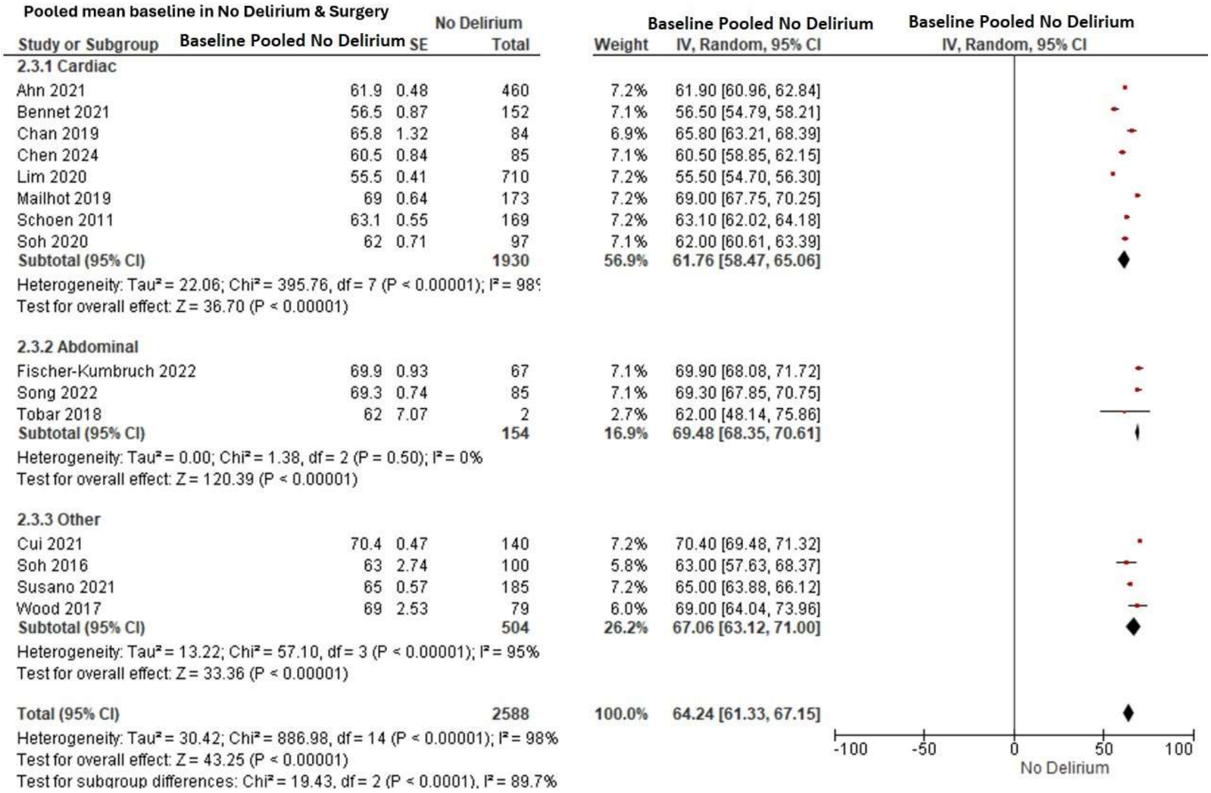

Figure S2. Mean baseline cerebral oxygen saturation values for (**a**) delirium and (**b**) non-delirium according to type of surgery

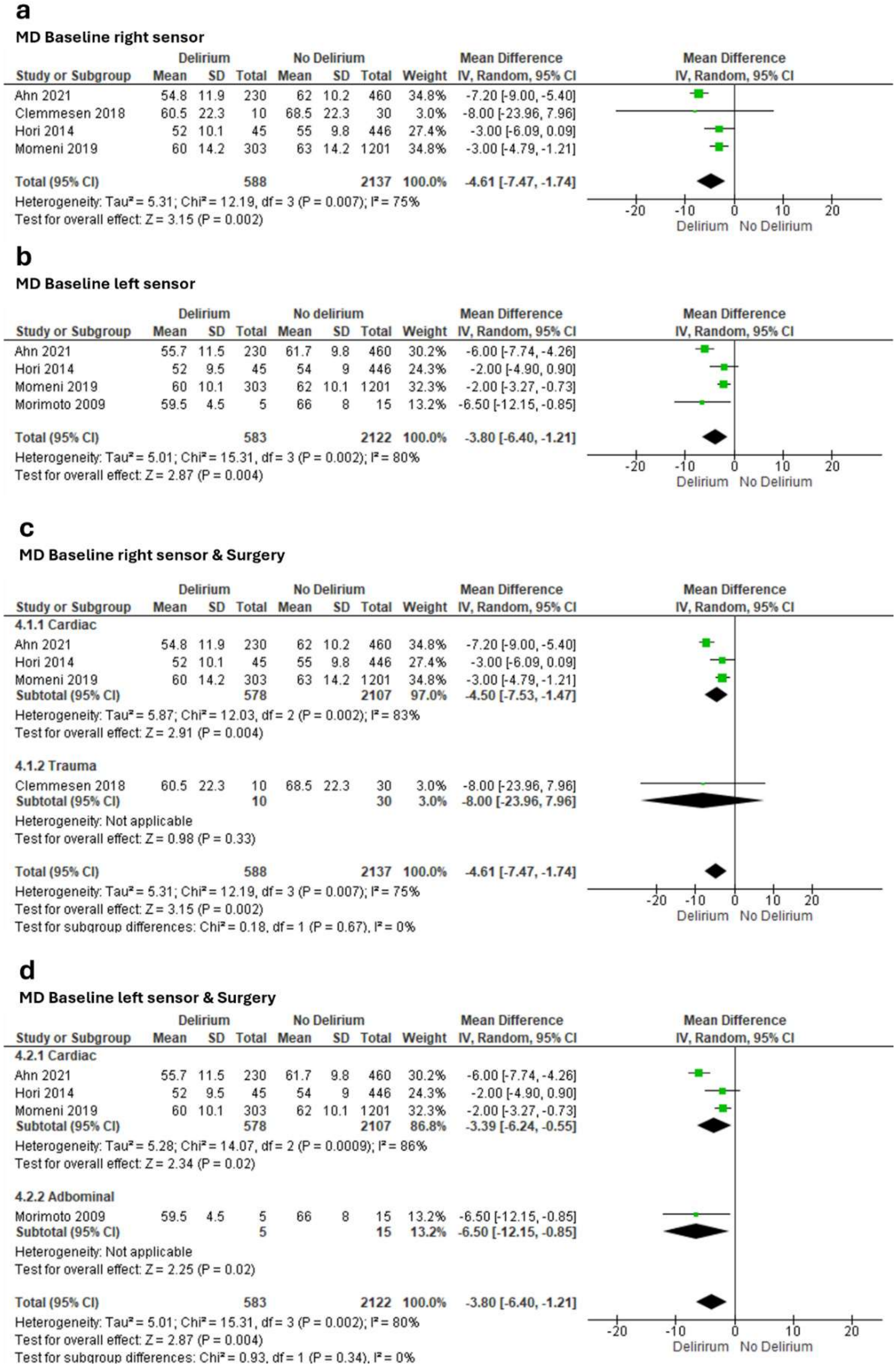

Figure S3. Meta-analysis of mean baseline cerebral oxygen saturation values–obtained via: (**a**) right sensor and (**b**) left sensor with the INVOS device; and (**c**) right sensor and (**d**) left sensor according to type of surgery–in people with and without subsequent delirium.

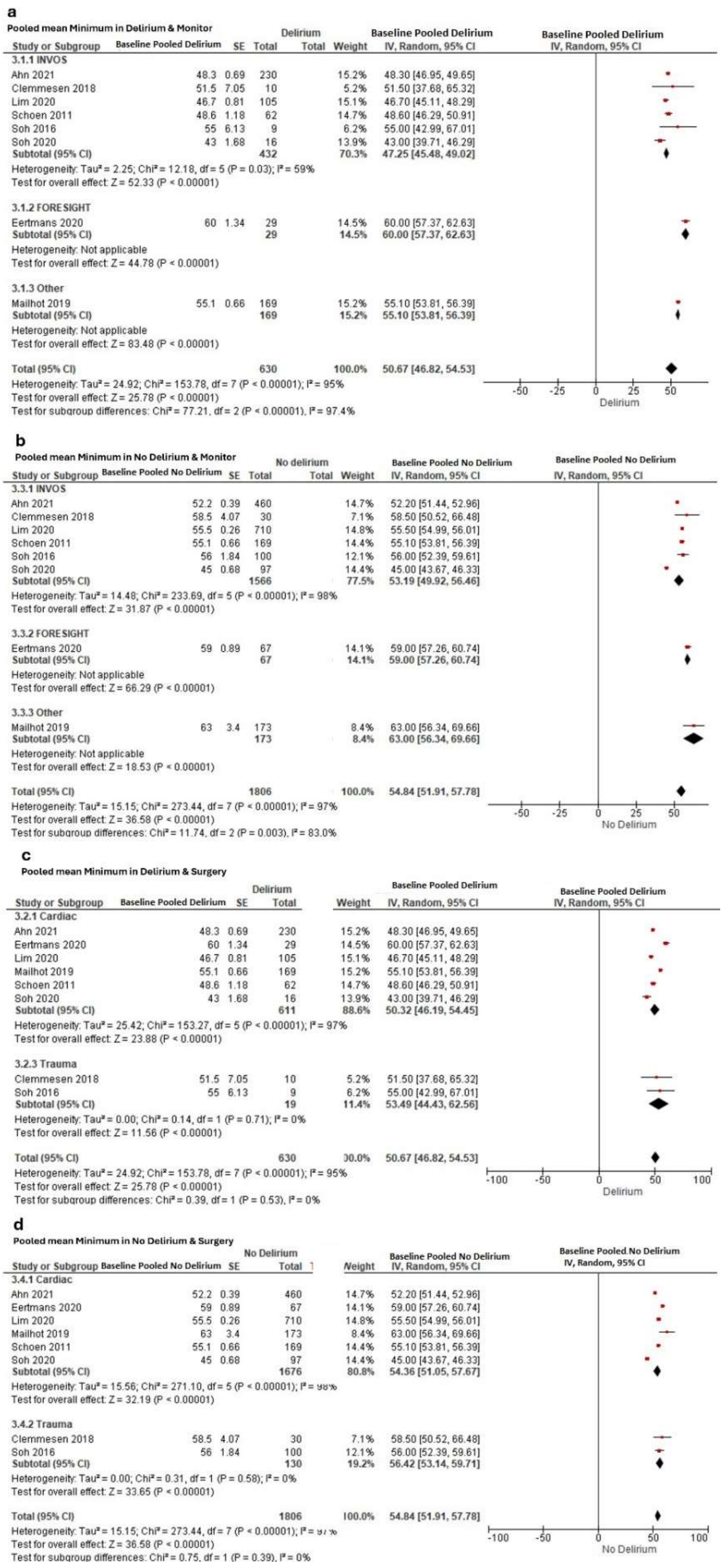

Figure S4. Mean minimum cerebral oxygen saturation values for: (**a**) delirium and (**b**) non-delirium according to monitor; and (**c**) delirium and (**d**) non-delirium according to surgery.

a

MD Minimum value according monitor

Study or Subgroup	Delirium Mean	SD	Total	No Delirium Mean	SD	Total	Weight	Mean Difference IV, Random, 95% CI
**1.10.1 INVOS**								
Ahn 2021	48.3	10.5	230	52.2	8.3	460	19.0%	-3.90 [-5.45, -2.35]
Clemmesen 2018	51.5	22.3	10	58.5	22.3	30	2.8%	-7.00 [-22.96, 8.96]
Lim 2020	46.7	8.3	105	55.5	6.8	710	18.9%	-8.80 [-10.46, -7.14]
Schoen 2011	48.6	9.3	62	55.1	8.6	169	17.2%	-6.50 [-9.15, -3.85]
Soh 2016	55	18.4	9	56	18.4	100	4.1%	-1.00 [-13.55, 11.55]
Soh 2020	43	6.7	16	45	6.7	97	15.4%	-2.00 [-5.54, 1.54]
**Subtotal (95% CI)**			**432**			**1566**	**77.4%**	**-5.33 [-8.10, -2.57]**
Heterogeneity: Tau² = 7.06; Chi² = 23.47, df = 5 (P = 0.0003); I² = 79%								
Test for overall effect: Z = 3.78 (P = 0.0002)								
**1.10.2 Foresight**								
Eertmans 2020	60	7.2	29	59	7.2	67	16.2%	1.00 [-2.14, 4.14]
**Subtotal (95% CI)**			**29**			**67**	**16.2%**	**1.00 [-2.14, 4.14]**
Heterogeneity: Not applicable								
Test for overall effect: Z = 0.62 (P = 0.53)								
**1.10.3 Other**								
Mailhot 2019	58	44.8	173	63	44.8	173	6.3%	-5.00 [-14.44, 4.44]
**Subtotal (95% CI)**			**173**			**173**	**6.3%**	**-5.00 [-14.44, 4.44]**
Heterogeneity: Not applicable								
Test for overall effect: Z = 1.04 (P = 0.30)								
**Total (95% CI)**			**634**			**1806**	**100.0%**	**-4.22 [-7.08, -1.36]**
Heterogeneity: Tau² = 10.57; Chi² = 40.19, df = 7 (P < 0.00001); I² = 83%								
Test for overall effect: Z = 2.89 (P = 0.004)								
Test for subgroup differences: Chi² = 9.06, df = 2 (P = 0.01), I² = 77.9%								

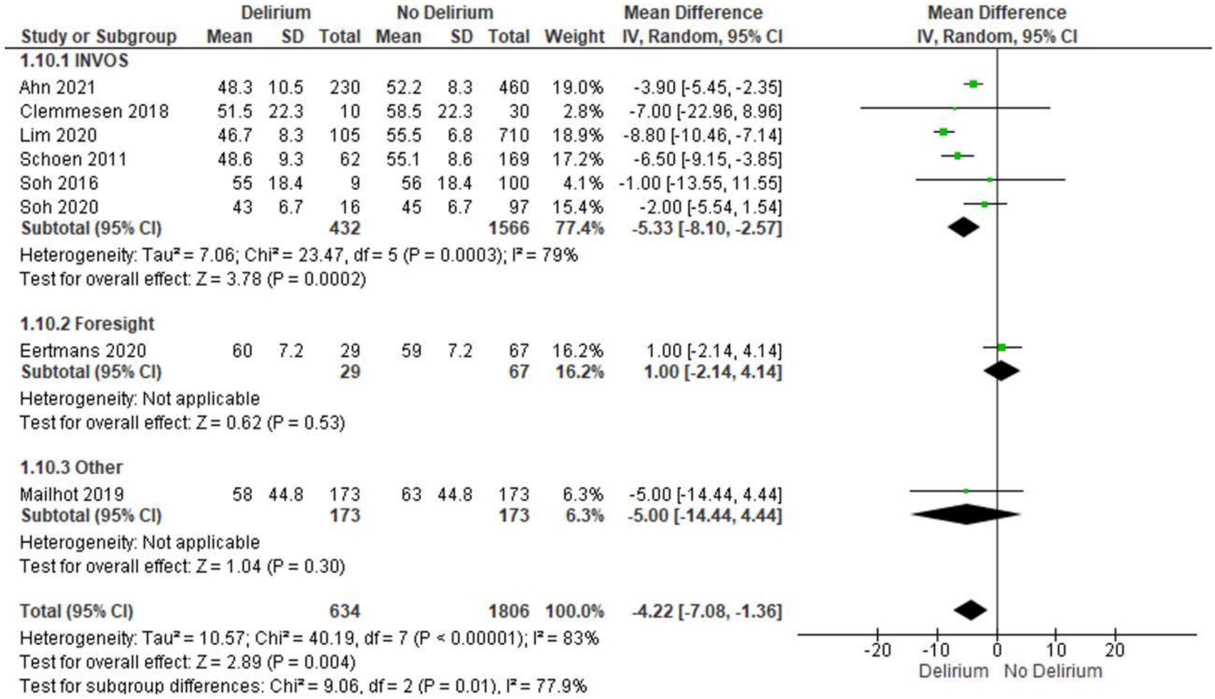

b

MD minimum & type of surgery

Study or Subgroup	Delirium Mean	SD	Total	No Delirium Mean	SD	Total	Weight	Mean Difference IV, Random, 95% CI
**3.5.1 Cardiac**								
Ahn 2021	48.3	10.5	230	52.2	8.3	460	19.0%	-3.90 [-5.45, -2.35]
Eertmans 2020	60	7.2	29	59	7.2	67	16.2%	1.00 [-2.14, 4.14]
Lim 2020	46.7	8.3	105	55.5	6.8	710	18.9%	-8.80 [-10.46, -7.14]
Mailhot 2019	58	44.8	173	63	44.8	173	6.3%	-5.00 [-14.44, 4.44]
Schoen 2011	48.6	9.3	62	55.1	8.6	169	17.2%	-6.50 [-9.15, -3.85]
Soh 2020	43	6.7	16	45	6.7	97	15.4%	-2.00 [-5.54, 1.54]
**Subtotal (95% CI)**			**615**			**1676**	**93.1%**	**-4.27 [-7.32, -1.23]**
Heterogeneity: Tau² = 11.24; Chi² = 39.71, df = 5 (P < 0.00001); I² = 87%								
Test for overall effect: Z = 2.75 (P = 0.006)								
**3.5.2 Trauma**								
Clemmesen 2018	51.5	22.3	10	58.5	22.3	30	2.8%	-7.00 [-22.96, 8.96]
Soh 2016	55	18.4	9	56	18.4	100	4.1%	-1.00 [-13.55, 11.55]
**Subtotal (95% CI)**			**19**			**130**	**6.9%**	**-3.29 [-13.16, 6.57]**
Heterogeneity: Tau² = 0.00; Chi² = 0.34, df = 1 (P = 0.56); I² = 0%								
Test for overall effect: Z = 0.65 (P = 0.51)								
**Total (95% CI)**			**634**			**1806**	**100.0%**	**-4.22 [-7.08, -1.36]**
Heterogeneity: Tau² = 10.57; Chi² = 40.19, df = 7 (P < 0.00001); I² = 83%								
Test for overall effect: Z = 2.89 (P = 0.004)								
Test for subgroup differences: Chi² = 0.03, df = 1 (P = 0.85), I² = 0%								

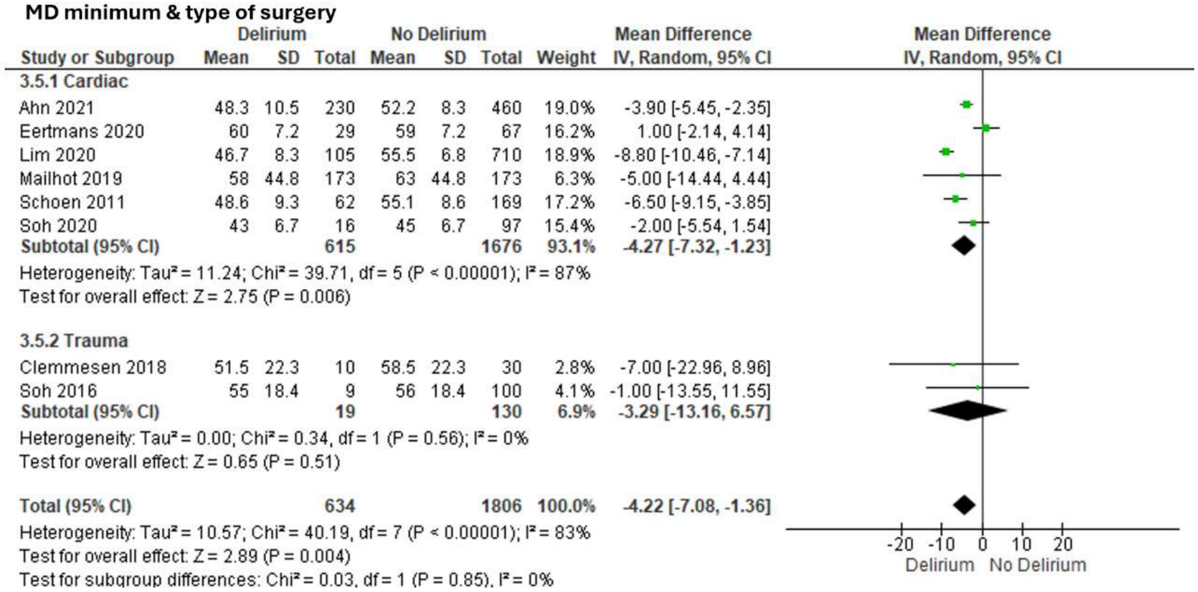

Figure S5. Meta-analysis of mean minimum cerebral oxygen saturation values obtained via bilateral sensors in people with and without subsequent delirium according to (**a**) monitor and (**b**) type of surgery.

Study or Subgroup	Delirium Mean	Delirium SD	Delirium Total	No Delirium Mean	No Delirium SD	No Delirium Total	Weight	Mean Difference IV, Random, 95% CI
**1.15.1 Continuous**								
Bennet 2021	54	10.7	14	56.5	10.7	152	4.1%	-2.50 [-8.36, 3.36]
Schoen 2011	58.1	7.7	62	63.1	7.2	169	9.4%	-5.00 [-7.20, -2.80]
Soh 2016	67	27.3	9	63	27.4	100	0.6%	4.00 [-14.63, 22.63]
Soh 2020	56	6	16	62	7	97	7.5%	-6.00 [-9.25, -2.75]
Tobar 2018	72	2	26	62	10	2	1.0%	10.00 [-3.88, 23.88]
**Subtotal (95% CI)**			**127**			**520**	**22.6%**	**-4.15 [-6.95, -1.35]**
Heterogeneity: Tau² = 3.37; Chi² = 6.37, df = 4 (P = 0.17); I² = 37%								
Test for overall effect: Z = 2.90 (P = 0.004)								
**1.15.2 Intervals**								
Ahn 2021	55.3	11.4	230	61.9	9.6	460	10.3%	-6.60 [-8.31, -4.89]
Chan 2019	67.4	12.1	24	65.8	12.1	84	4.4%	1.60 [-3.89, 7.09]
Chen 2024	58.2	6.9	47	60.5	7.7	85	8.8%	-2.30 [-4.86, 0.26]
Cui 2021	69.3	5.5	35	70.4	5.6	140	9.7%	-1.10 [-3.14, 0.94]
Fischer-Kumbruch 2022	64.8	8.33	13	69.9	7.6	67	5.1%	-5.10 [-9.98, -0.22]
Lim 2020	54.8	7.7	105	55.5	6.8	710	10.6%	-0.70 [-2.26, 0.86]
Song 2022	69.9	7.3	16	69.3	6.8	85	6.5%	0.60 [-3.26, 4.46]
Susano 2021	61	7.7	53	65	7.7	185	9.2%	-4.00 [-6.35, -1.65]
Wood 2017	63.5	15	19	69	22.5	79	2.4%	-5.50 [-13.87, 2.87]
**Subtotal (95% CI)**			**542**			**1895**	**67.1%**	**-2.54 [-4.51, -0.57]**
Heterogeneity: Tau² = 6.11; Chi² = 36.36, df = 8 (P < 0.0001); I² = 78%								
Test for overall effect: Z = 2.52 (P = 0.01)								
**1.15.3 Other**								
Mailhot 2019	66	8.4	173	69	8.4	173	10.2%	-3.00 [-4.77, -1.23]
**Subtotal (95% CI)**			**173**			**173**	**10.2%**	**-3.00 [-4.77, -1.23]**
Heterogeneity: Not applicable								
Test for overall effect: Z = 3.32 (P = 0.0009)								
**Total (95% CI)**			**842**			**2588**	**100.0%**	**-2.92 [-4.38, -1.47]**
Heterogeneity: Tau² = 4.58; Chi² = 47.02, df = 14 (P < 0.0001); I² = 70%								
Test for overall effect: Z = 3.94 (P < 0.0001)								
Test for subgroup differences: Chi² = 0.85, df = 2 (P = 0.65), I² = 0%								

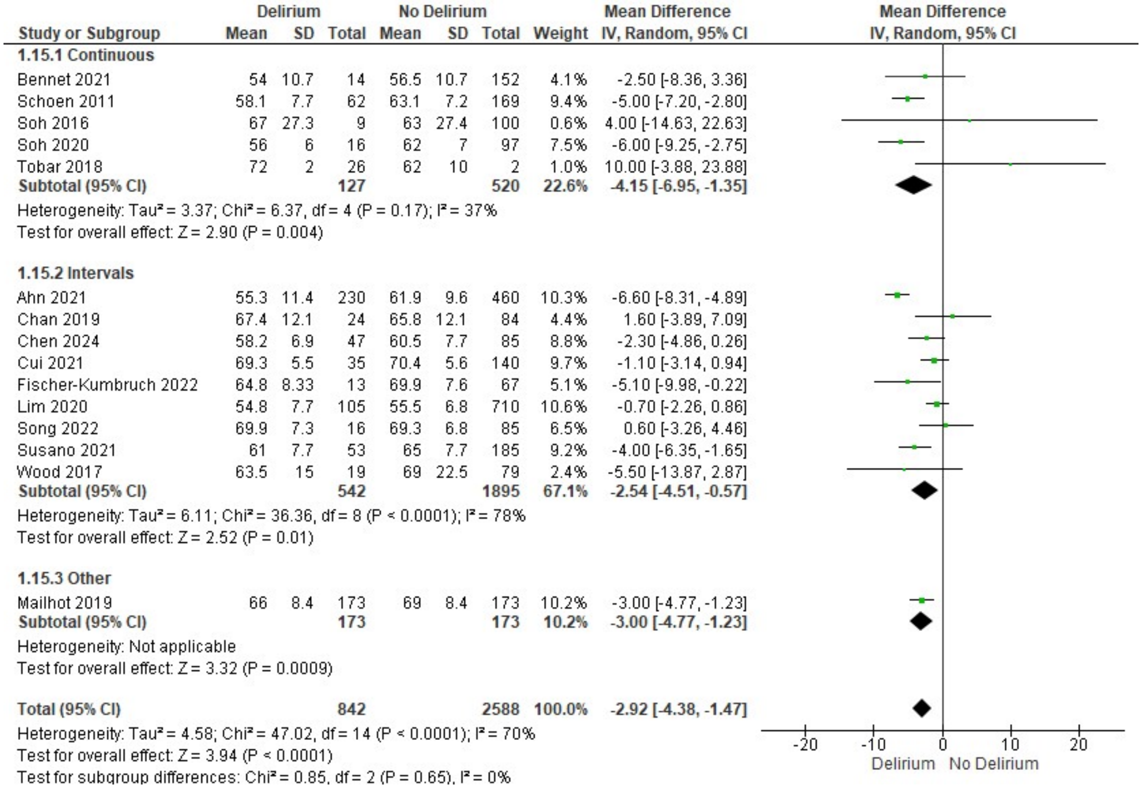

Figure S6. Meta-analysis of mean baseline cerebral oxygen saturation values obtained through bilateral sensors in people with and without subsequent delirium according to type of measurement.

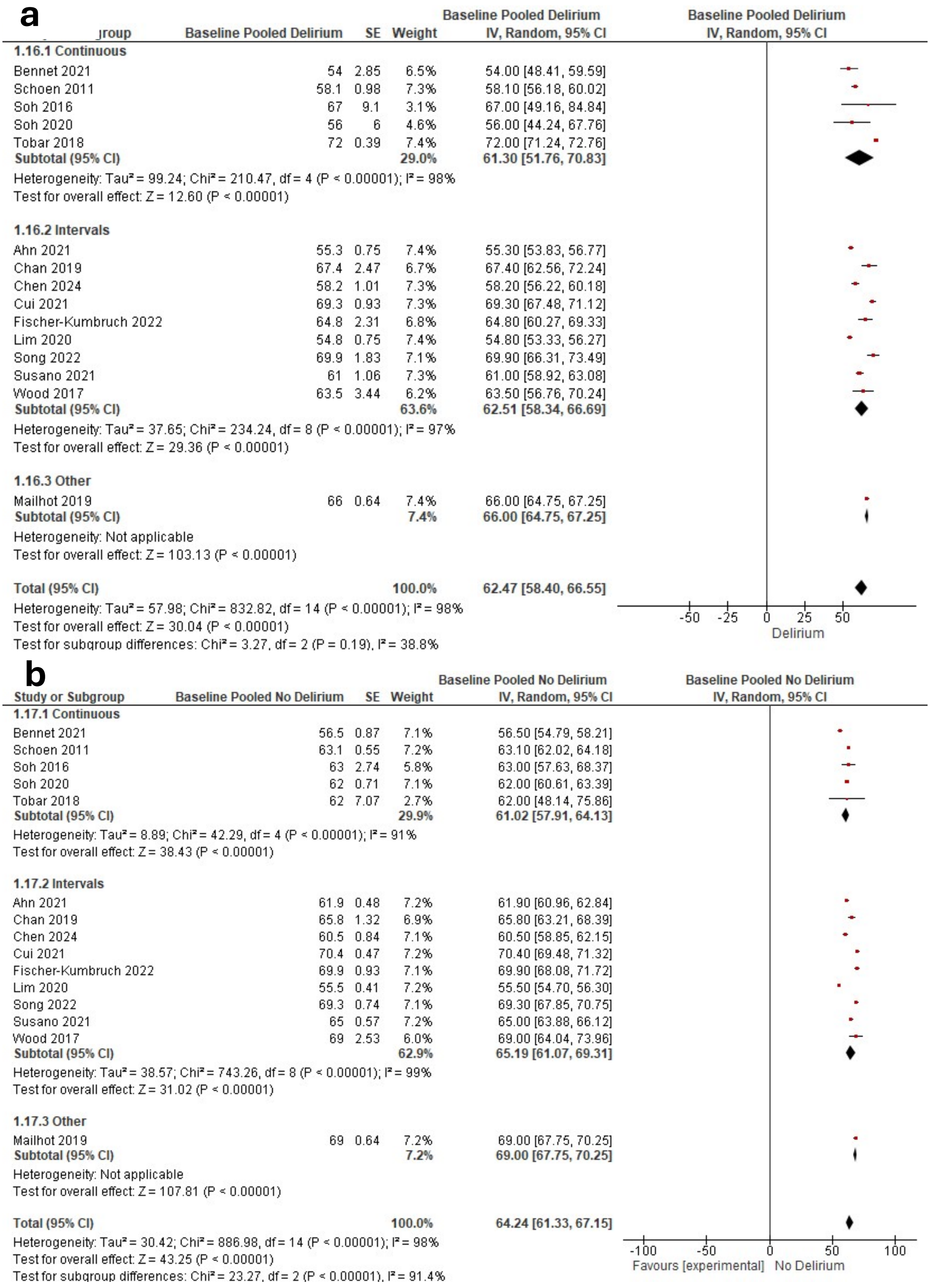

Figure S7. Mean baseline cerebral oxygen saturation values for (**a**) delirium and (**b**) non-delirium according to type of surgery

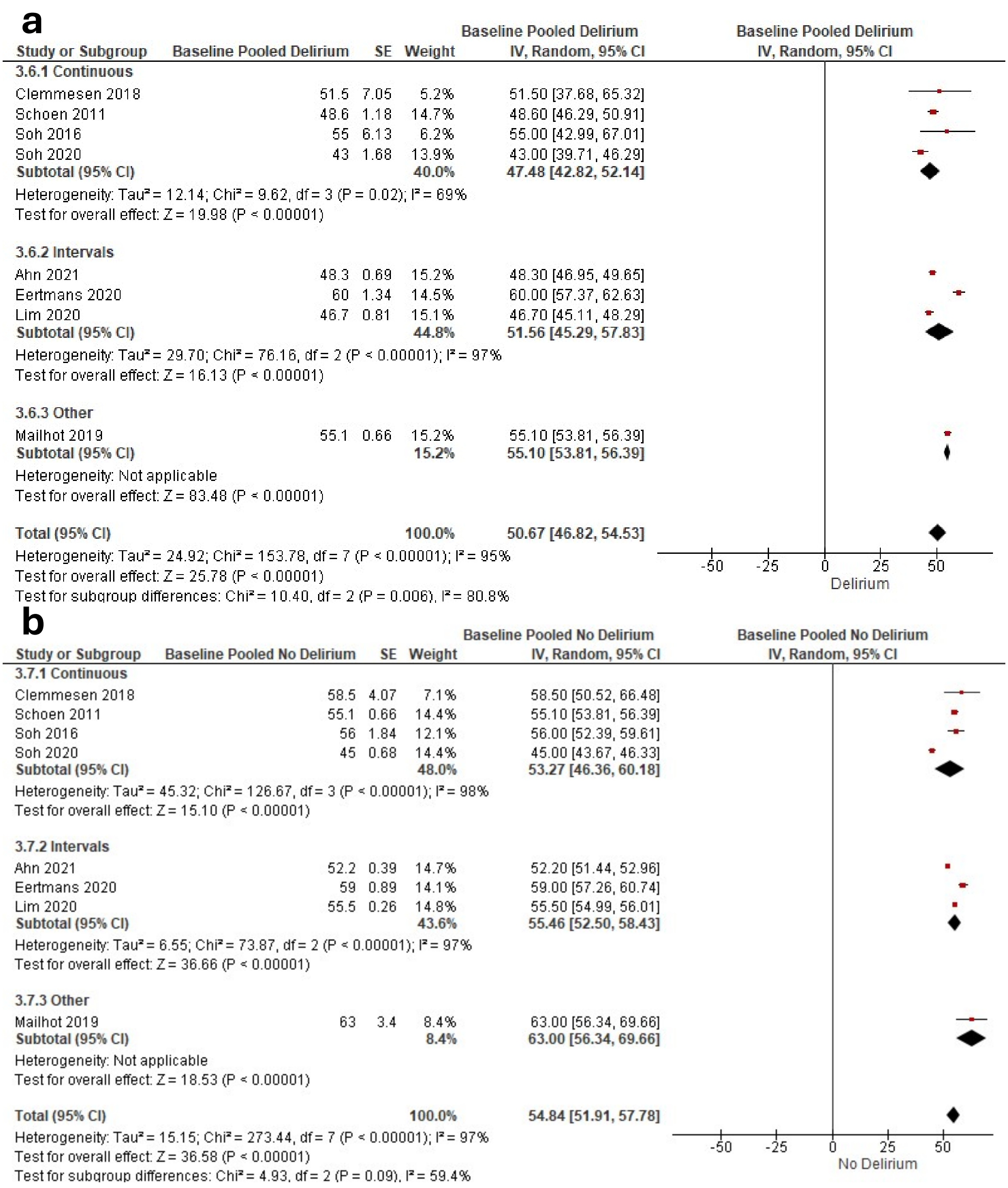

Figure S8. Mean minimum cerebral oxygen saturation values for: (**a**) delirium and (**b**) non-delirium according to type of measurement

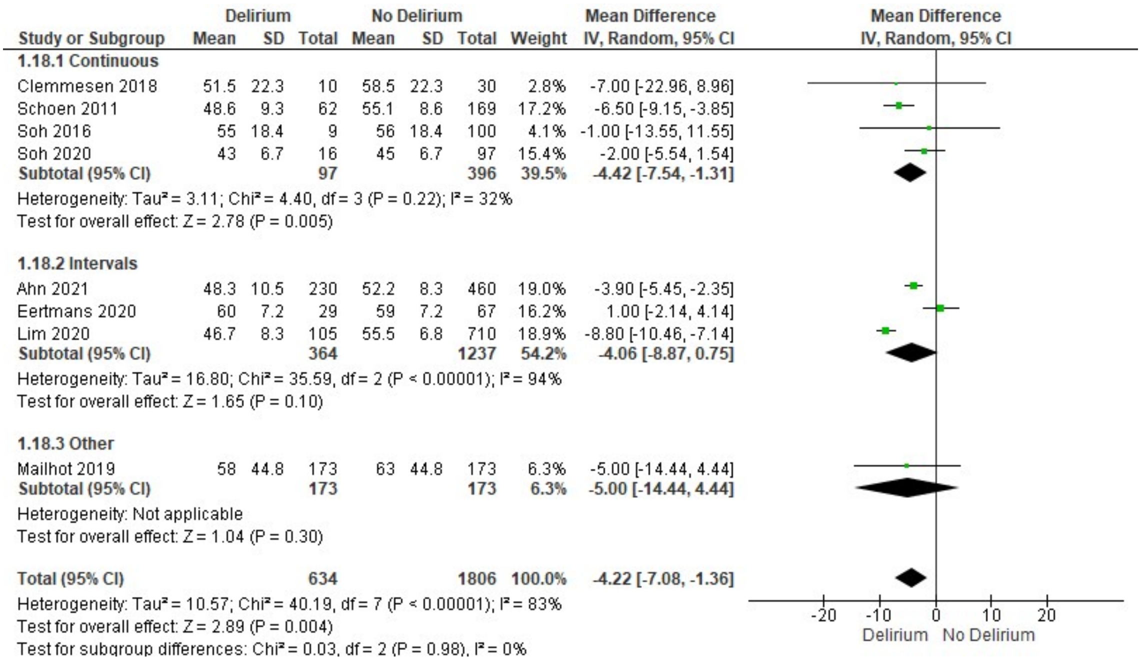

Study or Subgroup	Delirium Mean	Delirium SD	Delirium Total	No Delirium Mean	No Delirium SD	No Delirium Total	Weight	Mean Difference IV, Random, 95% CI
**1.18.1 Continuous**								
Clemmesen 2018	51.5	22.3	10	58.5	22.3	30	2.8%	-7.00 [-22.96, 8.96]
Schoen 2011	48.6	9.3	62	55.1	8.6	169	17.2%	-6.50 [-9.15, -3.85]
Soh 2016	55	18.4	9	56	18.4	100	4.1%	-1.00 [-13.55, 11.55]
Soh 2020	43	6.7	16	45	6.7	97	15.4%	-2.00 [-5.54, 1.54]
**Subtotal (95% CI)**			**97**			**396**	**39.5%**	**-4.42 [-7.54, -1.31]**
Heterogeneity: Tau² = 3.11; Chi² = 4.40, df = 3 (P = 0.22); I² = 32%								
Test for overall effect: Z = 2.78 (P = 0.005)								
**1.18.2 Intervals**								
Ahn 2021	48.3	10.5	230	52.2	8.3	460	19.0%	-3.90 [-5.45, -2.35]
Eertmans 2020	60	7.2	29	59	7.2	67	16.2%	1.00 [-2.14, 4.14]
Lim 2020	46.7	8.3	105	55.5	6.8	710	18.9%	-8.80 [-10.46, -7.14]
**Subtotal (95% CI)**			**364**			**1237**	**54.2%**	**-4.06 [-8.87, 0.75]**
Heterogeneity: Tau² = 16.80; Chi² = 35.59, df = 2 (P < 0.00001); I² = 94%								
Test for overall effect: Z = 1.65 (P = 0.10)								
**1.18.3 Other**								
Mailhot 2019	58	44.8	173	63	44.8	173	6.3%	-5.00 [-14.44, 4.44]
**Subtotal (95% CI)**			**173**			**173**	**6.3%**	**-5.00 [-14.44, 4.44]**
Heterogeneity: Not applicable								
Test for overall effect: Z = 1.04 (P = 0.30)								
**Total (95% CI)**			**634**			**1806**	**100.0%**	**-4.22 [-7.08, -1.36]**
Heterogeneity: Tau² = 10.57; Chi² = 40.19, df = 7 (P < 0.00001); I² = 83%								
Test for overall effect: Z = 2.89 (P = 0.004)								
Test for subgroup differences: Chi² = 0.03, df = 2 (P = 0.98), I² = 0%								

Figure S9. Meta-analysis of mean minimum cerebral oxygen saturation values obtained via bilateral sensors in people with and without subsequent delirium according to type of measurement.

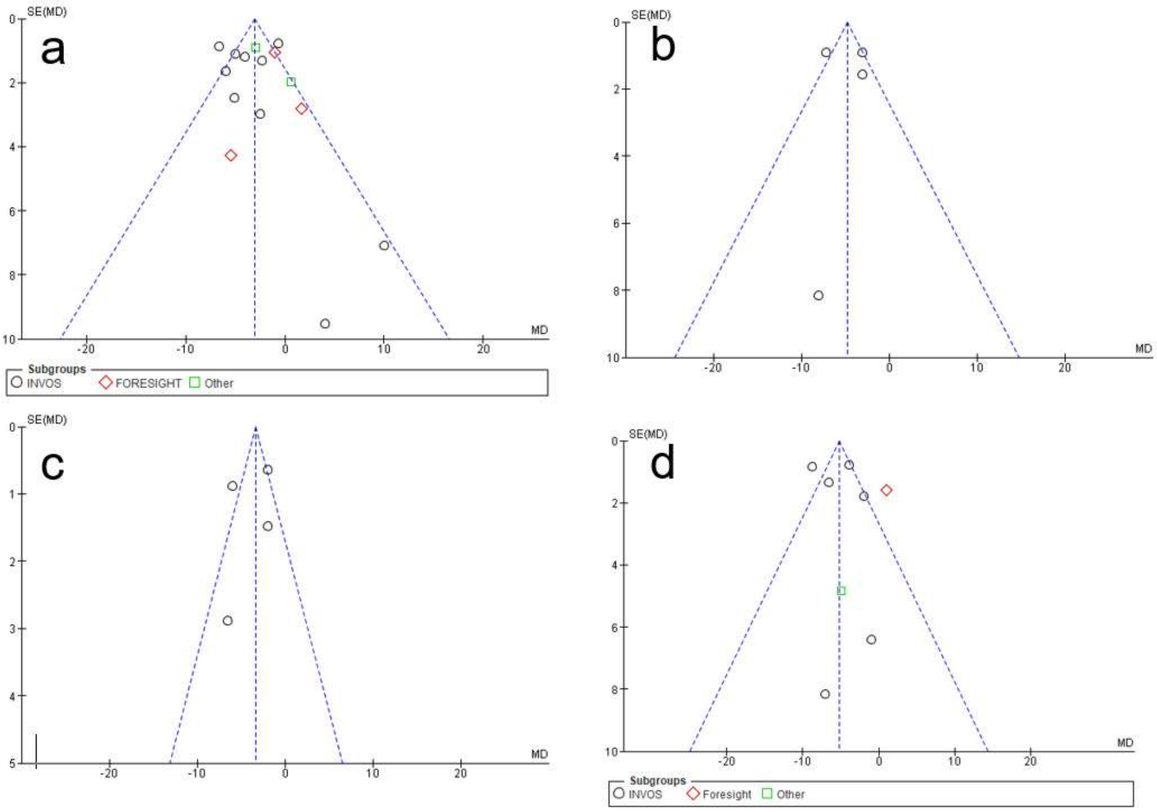

Figure S10. Funnel plot of (**a**) subgroup baseline mean difference according to device baseline, (**b**) mean difference according to device right sensor, (**c**) baseline mean difference according to device left sensor and (**d**) minimum mean difference according to device.
